# Identifying the risk regions of house break‐ins caused by Tibetan brown bears (*Ursus arctos pruinosus*) in the Sanjiangyuan region, China

**DOI:** 10.1002/ece3.5835

**Published:** 2019-12-08

**Authors:** Yunchuan Dai, Charlotte E. Hacker, Yuguang Zhang, Wenwen Li, Jia Li, Yu Zhang, Gongbaocairen Bona, Haodong Liu, Ye Li, Yadong Xue, Diqiang Li

**Affiliations:** ^1^ Research Institute of Forest Ecology, Environment and Protection Chinese Academy of Forestry Beijing China; ^2^ Key Laboratory of Biodiversity Conservation State Forestry and Grassland Administration Beijing China; ^3^ Department of Biological Sciences Duquesne University Pittsburgh PA USA; ^4^ Key Laboratory for Biodiversity Science and Ecological Engineering Ministry of Education College of Life Sciences Beijing Normal University Beijing China; ^5^ Institute of Desertification Studies Chinese Academy of Forestry Beijing China; ^6^ Qilian Mountain National Park Qinghai Administration Xining China; ^7^ The People's Government of Duocai Township Zhiduo China; ^8^ Research Institute of Forest Resource Information Techniques Chinese Academy of Forestry Beijing China

**Keywords:** bear damage, house break‐ins, risk assessment, risk diffusion path, Sanjiangyuan region, *Ursus arctos pruinosus*

## Abstract

Damage to homesteads by brown bears (*Ursus arctos*) has become commonplace in Asia, Europe, and the Americas. Science‐based solutions for preventing damages can contribute to the establishment of mechanisms that promote human–bear coexistence. We examined the spatial distribution patterns of house break‐ins by Tibetan brown bears (*U. a. pruinosus*) in Zhiduo County of the Sanjiangyuan region in China. Occurrence points of bear damage were collected from field surveys completed from 2017 to 2019. The maximum entropy (MaxEnt) model was then used to assess house break‐in risk. Circuit theory modeling was used to simulate risk diffusion paths based on the risk map generated from our MaxEnt model. The results showed that (a) the total risk area of house break‐ins caused by brown bears was 11,577.91 km^2^, accounting for 29.85% of Zhiduo County, with most of the risk areas were distributed in Sanjiangyuan National Park, accounting for 58.31% of the total risk area; (b) regions of alpine meadow located in Sanjiangyuan National Park with a high human population density were associated with higher risk; (c) risk diffusion paths extended southeast to northwest, connecting the inside of Sanjiangyuan National Park to its outside border; and (d) eastern Suojia, southern Zhahe, eastern Duocai, and southern Jiajiboluo had more risk diffusion paths than other areas examined, indicating higher risk for brown bear break‐ins in these areas. Risk diffusion paths will need strong conservation management to facilitate migration and gene flow of brown bears and to alleviate bear damage, and implementation of compensation schemes may be necessary in risk areas to offset financial burdens. Our analytical methods can be applied to conflict reduction efforts and wildlife conservation planning across the Qinghai–Tibet Plateau.

## Graphical abstract

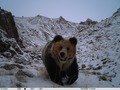

## 1 INTRODUCTION

Anthropogenic influences have affected every ecosystem on earth, with demands and pressures on wildlife resources steadily increasing (Samojlik et al., 2018; Strum, 2010). Over‐exploitation of natural resources reduces, fragments, and isolates wildlife habitat across landscapes, and threatens numerous endangered animal species (Fahrig, 2003; Rushton, Wood, Lurz, & Koprowski, 2006). As a result, these species must capitalize on novel human resources to survive, causing conflict between humans and wildlife (Samojlik et al., 2018; Soofi, Qashqaei, Aryal, & Coogan, 2018; Strum, 2010). These instances are exacerbated when guided conservation policies facilitate wildlife population recovery, but existing habitat is unable to support these increased numbers, increasing human‐wildlife conflict (HWC; Messmer, 2000).

Human‐wildlife conflict results in negative outcomes for humans or their resources, and wildlife and their habitats (Can, D'Cruze, Garshelis, Beecham, & Macdonald, 2014; Lamichhane et al., 2018; van Eeden et al., 2018). Conflict types include house break‐ins (Dai, Li, et al., 2019; Dai, Xue, et al., 2019), human injury and death (Dai, Xue, et al., 2019; Pozsgai, 2017; White & Ward, 2010), livestock depredation (Dai, Xue, et al., 2019; Meinecke et al., 2018; Peterson, Birckhead, Leong, Peterson, & Peterson, 2010; van Eeden et al., 2018), crop raiding (Liu et al., 2011), and disease transmission (DeCandia, Dobson, & vonHoldt, 2018). Consequences for wildlife include removal, retaliatory killing, and reduced tolerance for coexistence, which can lead to population decline, fragmentation (Proctor et al., 2012), and elevated conservation concerns (Dickman, 2010; Li, Yin, Wang, Jiagong, & Lu, 2013; van Eeden et al., 2018). Attacks on humans by endangered animals that are legally protected are especially controversial (Prasad, Kumar, Raj, Michael, & Bi‐Song, 2016). This is because if human beings hurt these animals, they would be punished by law. But if these animals hurt humans, then humans can only get a little financial compensation. Human–wildlife coexistence strategies are influenced by many factors, including religion, cultural, and economic value of wildlife products, and the financial losses stemming from the conflict, making them difficult to manage (Aryal, Morley, & McLean, 2018; Dickman, 2010; Li et al., 2013). Previous studies have linked the increase of HWC and its associated economic loss in many countries, including China (Aryal, Brunton, Ji, Barraclough, & Raubenheimer, 2014; Karamanlidis, Sanopoulos, Georgiadis, & Zedrosser, 2011; Li et al., 2013, 2018; Liu et al., 2011; Rigg et al., 2011).

**Figure 1:**
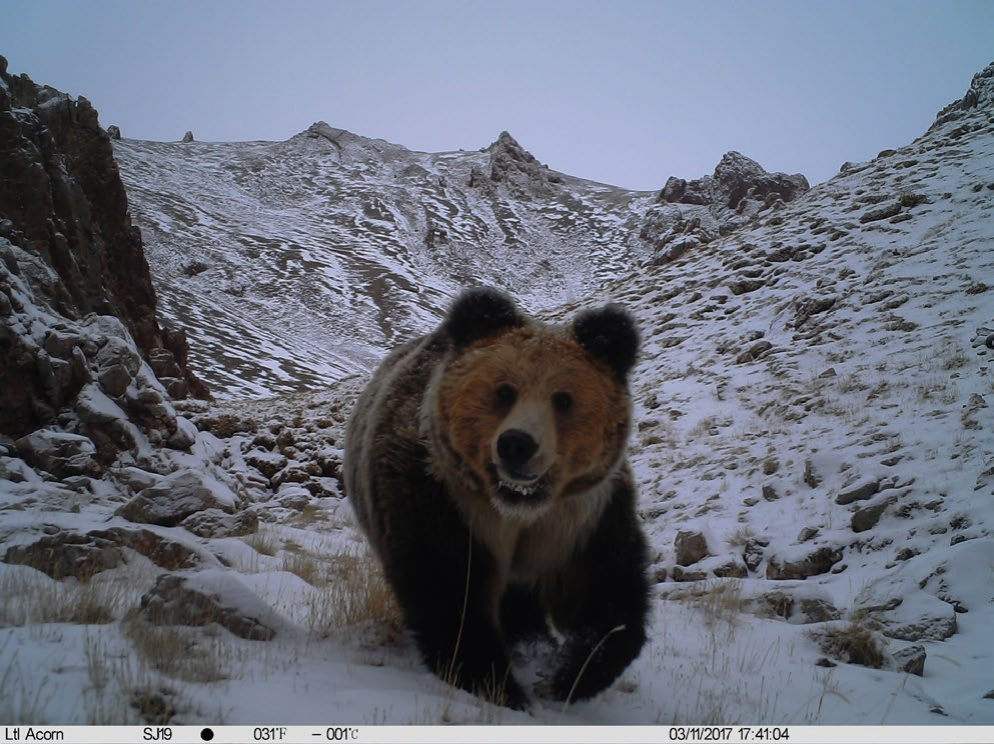
Tibetan brown bear (*Ursus arctos pruinosus*) captured by camera trapping in the Yangtze River Zone of Sanjiangyuan National Park, China

The Tibetan brown bear (*Ursus arctos pruinosus*; Figure 1), also known as the Tibetan blue bear, is a rare subspecies of brown bear living at high altitudes in close proximity to humans in Central Asia (Dai, Hacker, et al., 2019; Dai, Li, et al., 2019; Dai, Xue, et al., 2019; Xu et al., 2006). The species population estimate is between 5,000 and 6,000 individuals (Wu, 2014). Due to effective conservation policies and reduced hunting in the Sanjiangyuan region, Tibetan brown bears are becoming more abundant each year (Yan et al., 2019). However, humans have overexploited large‐scale grasslands to meet growing demands in the past few decades, resulting in a declining habitat quality and a highly fragmented distribution of brown bears (Nawaz, Martin, & Swenson, 2014). Large areas of alpine meadow, a preferred habitat of the brown bear, have been degraded due to overgrazing in the Sanjiangyuan region, disrupting the regular migration paths of brown bears and increasing the overlap between species' distribution and herders' living areas (Dai, Hacker, et al., 2019). The attempt to take novel migration routes coupled with increased overlap has caused an increase in the number of human–bear conflicts, severely elevating the degree of bear damage. Moreover, the high altitude of the Sanjiangyuan region limits the number of plant food species rich in sugar and fat energy that can cultivate there. Thus, brown bears rely on digging pikas (*Ochotona curzoniae*) and marmots (*Marmota himalayana*) for food, but these resources can take a substantial amount of energy to procure (Wu, 2014). The presence of readily available and high‐energy food sources in herders' residential areas serve as a much more efficient foraging strategy, but increase the risk of brown bears damaging herders' property and threatening herders' personal safety (Dai, Xue, et al., 2019).

In China, conflict between humans and Tibetan brown bears is prominent among HWC events (Dai, Li, et al., 2019; Dai, Xue, et al., 2019). These events can be extreme with reports of brown bears attacking people and preying on livestock (Aryal, Hopkins, Raubenheimer, & Brunton, 2012; Dai, Li, et al., 2019; Dai, Xue, et al., 2019; Han et al., 2018; Yan et al., 2019). House break‐ins by Tibetan brown bears started in 1998 when herders started to build winter homes in the Sanjiangyuan region of China. According to the Wildlife Damage Compensation Program (WDCP) of Qinghai Forestry and Grassland Administration, wildlife damage in Qinghai Province between 2014 and 2017 resulted in financial compensation of approximately $4.03 million USD, of which $1.69 million USD (41.94%) was caused by brown bear (data provided by the WDCP). Brown bears damage home doors, windows, furniture, and daily supplies (Dai, Li, et al., 2019; Dai, Xue, et al., 2019; Han et al., 2018; Wu, 2014). In addition, they threaten local herders' physical safety (Aryal et al., 2014; Aryal, Raubenheimer, et al., 2012; Dai, Li, et al., 2019; Dai, Xue, et al., 2019; Han et al., 2018; Wu, 2014). Between 2014 and 2017, 14 herders were attacked by brown bears in Qinghai Province, injuring five people and killing nine (data provided by the WDCP). Bear damage has become an increasingly urgent and important issue for China to resolve but is complicated by the need to preserve and protect the species (Dai, Li, et al., 2019; Dai, Xue, et al., 2019). To foster human–bear coexistence and implement spatially differentiated management of risk areas, it is imperative to better understand brown bear ecology, determine the relationship between risk areas and their surrounding environment, and to understand the spatial distribution characteristics associated with incidents of brown bear damage.

Our research used maximum entropy (MaxEnt) modeling to identify regions at risk of house break‐ins where Tibetan brown bears are currently distributed and to predict future risks. We used the circuit theory model based on the random walk theory to simulate the risk diffusion paths of Tibetan brown bears within Zhiduo County of Sanjiangyuan region. We aimed to achieve the following goals: (a) explore how ecological environment variables are linked to risk areas and analyze the spatial distribution characteristics of risk regions; (b) identify risk diffusion paths and analyze connections between Tibetan brown bears located in and outside the Sanjiangyuan National Park; (c) combine the results of (a) and (b) to discuss the risk areas and risk diffusion paths; and (d) suggest mitigation strategies in accordance to differing risk levels and diffusion paths. The purpose of this study is to provide scientific support for the management of brown bear damage in Sanjiangyuan National Park and to provide feasible recommendations for the protection of Tibetan brown bears and herder livelihoods.

## 2 MATERIAL AND METHODS

### 2.1 Study area

**Figure 2:**
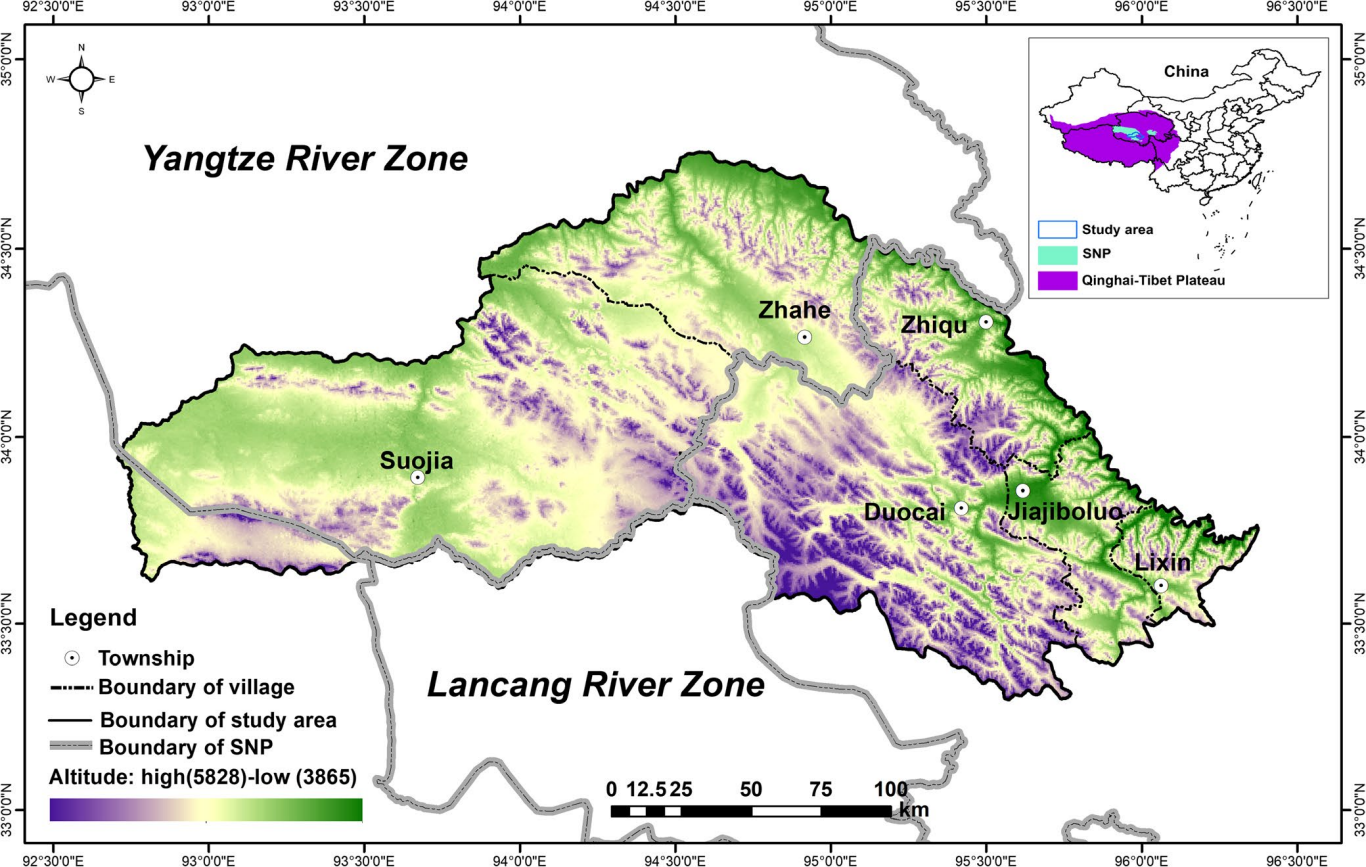
Location of Zhiduo County of Sanjiangyuan region, Qinghai–Tibet Plateau. The study area excludes Hoh Xil National Nature Reserve and only includes Suojia, Zhahe, Duocai, Zhiqu, Lixin and Jiajiboluo. Among them, Suojia and Zhahe are encompassed by the Yangtze River Zone of Sanjiangyuan National Park, Duocai and Suojia border the Lancang River Zone of Sanjiangyuan National Park (SNP)

Zhiduo County of Sanjiangyuan region (between 92.7066° and 96.3751°E, 33.2948° and 34.7546°N; excluding Hoh Xil National Nature Reserve; 38,793.4 km^2^) has an average altitude of 4,500 m and is located on the central part of the Qinghai–Tibet Plateau in China (Figure 2). The area is dominated by alpine meadow vegetation, accounting for 50.84% of the study area. The weather is typically dry and cold with the annual average temperature ranging from −0.3°C to −6°C and the annual precipitation consisting mostly of snow fall ranging from 150 to 420 mm. Zhiduo County is rich in wildlife species. Representative ungulates include Przewalski's gazelle (*Procapra przewalskii*), Tibetan antelope (*Pantholops hodgsonii*), Tibetan gazelle (*Procapra picticaudata*), wild yak (*Bos mutus*), Kiang (*Equus kiang*), and blue sheep (*Pseudois nayaur*). Carnivores include snow leopard (*Panthera uncia*), Tibetan brown bear, gray wolf (*Canis lupus*), Tibetan fox (*Vulpes ferrilata*), and dhole (*Cuon alpinus*) (data provided by the Sanjiangyuan National Park administration; http://sjy.qinghai.gov.cn/). Zhiduo has 68 pastoral communities spread across 5 townships (Suojia, Zhahe, Duocai, Zhiqu, and Lixin) and 1 town (Jiajiboluo) with 34,236 inhabitants. Tibetans account for more than 98.35% of residents, with the remainder being Han, Hui people, and others (data provided by the government of Zhiduo County; http://www.zhiduo.gov.cn/). Among them, Suojia and Zhahe are encompassed by the Yangtze River Zone of Sanjiangyuan National Park, Duocai and Suojia border the Lancang River Zone of Sanjiangyuan National Park (Figure 2).

### 2.2 Occurrence records

All appropriate permits and permissions for research involving human subjects were acquired prior to study start. An Institutional Review Board exemption was granted as no identifying information was collected during the interview. Data on bear damage occurrences were compiled from field surveys conducted at different times between 2017 and 2019 in various localities of Zhiduo County, Qinghai Province. Presence points of bear damage were collected by conducting household surveys and semi‐structured interviews during field surveys carried out as part of other research projects (Dai, Hacker, et al., 2019; Dai, Li, et al., 2019; Dai, Xue, et al., 2019). Coordinates of bear damage were recorded with GPS. Incidents primarily included house break‐ins (including damage of doors, windows, furniture, and daily supplies) and livestock depredation in domestic animal enclosures. In total, we interviewed 255 households in Zhiduo County and collected 239 GPS coordinates of bear damage. Among them, 228 GPS coordinates consisted of house break‐ins, and 11 for both house break‐ins and penned livestock depredation. We attributed each accident location as an occurrence point. Only 16 households did not experience bear damage. In order to reduce autocorrelation, we filtered occurrence points by randomly selecting one point in each grid of 1 km^2^ according to model optimization recommendations and previous studies (Aryal et al., 2016; Dai, Hacker, et al., 2019; Li, Liu, Xue, Zhang, & Li, 2017; Phillips, Anderson, & Schapire, 2006).

### 2.3 Environmental variables

We selected environmental variables based on geographic environmental characteristics, human disturbance, brown bear ecology, and those used in previously published literature (Dai, Hacker, et al., 2019; Li et al., 2018; Wu, 2014). Environmental variables were categorized into three groups: (a) geographic environmental factors, such as elevation, slope, aspect, distance to rivers, density of river distribution, distance to lakes, and density of lake distribution; (b) human factors, such as human population density, human influence index; and (c) factors associated with brown bear ecology, such as distance to the Sanjiangyuan National Park, land use type, and Normalized Difference Vegetation Index (NDVI). Variables considered were not only related to anthropogenic disturbance, but also to brown bear distribution. For example, the distance to the Sanjiangyuan National Park is an important variable for assessing brown bear distribution because the park provides important habitat and refugia for brown bears (Dai, Hacker, et al., 2019; Dai, Li, et al., 2019; Dai, Xue, et al., 2019); land use type and NDVI determine the richness of natural food source of the brown bear.

Elevation, aspect, and slope were derived from the ASTER GDEM V2 digital elevation model (at 30 m resolution; http://www.gscloud.cn/). Normalized Difference Vegetation Index (NDVI) and human population density were obtained from the Resource and Environment Data Cloud Platform (NDVI in 2018; human population density in 2015; at 1 km resolution; http://www.resdc.cn/). Human Influence Index (HII) was obtained from the Socioeconomic Data and Applications Center, NASA (Last of the Wild, v2; at 1 km resolution; http://sedac.ciesin.columbia.edu/). HII represents anthropogenic impacts (1995–2004), calculated by integrating human accessibility, human land use, and human population pressure.

Land use/land cover data of the study site were obtained by interpreting 2017 Landsat 8 OLI (at 30 m resolution; U.S. Geological Survey; https://www.usgs.gov/) and adopting a 1:50,000 digital elevation model (DEM) as a reference control image to correct for geometric biases by using ENVI 5.1 (ESRI Inc.). An RMS error <1 indicates that the land use/land cover data fulfills the precision standards of research. Land use/land cover data were organized into 12 categories: (1) coniferous forest, (2) bush, (3) alpine meadow, (4) alpine steppe, (5) swamp, (6) water body, (7) river bed, (8) bare rock, (9) desert, and (10) other.

### 2.4 Variable standardization and selection

All spatial variables were resampled to 500 m resolution (Li et al., 2019) and unified projection coordinate system (Clarke_1866_Albers) in ArcGIS 10.1 (ESRI Inc.). The correlation coefficient of variables was computed by using the tool of Band Collection Statistics (BCS) in ArcGIS 10.1. In order to identify the key variables affecting estimation model of human–bear conflict risk, variables were screened in a series of three steps. First, the multicollinearity of variables was reduced by eliminating correlation variables where |*r*| > 0.6 (Li et al., 2018; Appendix S1). Second, we introduced the remaining variables to model and removed those with no contribution rates. Third, the most influential variables based on contribution rates obtained from the output of the first model were selected, and the model repeated (Dai, Hacker, et al., 2019).

### 2.5 Modeling risk distributions of house break‐ins

There were 16 households that reported they did not experience bear damage. The logistic regression model is more appropriate than the presence‐only maximum entropy (MaxEnt) model, because the risk distribution map is better‐informed with both presence and absence data than with presence data alone (Miller, 2015). However, these 16 households were primarily concentrated in residential areas with a higher density of humans and thus greater human interference; therefore, these occurrence points cannot objectively represent the environmental characteristics of the houses that were not damaged by brown bears. Hence, we selected the MaxEnt model to predict risk distributions of house break‐ins (Dai, Hacker, et al., 2019; Li et al., 2018). The parameters of MaxEnt model were set to 25% for random test percentage and 1 regularization multiplier (Aryal et al., 2016; Su et al., 2018). We ran 15 replicates and preformed a cross‐validation (Dai, Hacker, et al., 2019; Phillips et al., 2006; Vedel‐Sørensen, Tovaranonte, Bøcher, Balslev, & Barfod, 2013). Percent contribution was used to estimate the importance of variables. Risk maps were calculated using the logistic output of MaxEnt, and the logical risk index (RI) is from the lowest “0” to the highest “1.” We classified the risk maps into four categories including “high risk” (RI ≥ 0.6), “medium risk” (0.4 ≤ RI ＜ 0.6), “low risk” (0.2 ≤ RI ＜ 0.4), and “nonrisk” (RI ＜ 0.2; Yang, Kushwaha, Saran, Xu, & Roy, 2013; Convertino, Muñoz‐Carpena, Chu‐Agor, Kiker, & Linkov, 2014; Ansari & Ghoddousi, 2018).

We evaluated MaxEnt model performance by using the area under the receiver operating characteristic curve (AUC) (Su et al., 2018). AUC is an independent threshold value to verify the accuracy of model outputs, and its value ranges from 0 to 1. When AUC is closer to 1, the accuracy of the model is higher (Araujo, Pearson, Thuiller, & Erhard, 2005; Dai, Hacker, et al., 2019; Phillips et al., 2006).

### 2.6 Predicting risk diffusion paths

Circuit model is based on circuit theory, predicting the movement of random walkers between source and target cells across a landscape by electricity patterns (Dai, Hacker, et al., 2019; McRae & Beier, 2007; McRae, Shah, & Mohapatra, 2013; Walpole, Bowman, Murray, & Wilson, 2012). We simulated risk diffusion paths for brown bears based on the risk map from our MaxEnt model by using Circuitscape software 4.0 (https://circuitscape.org/). The model mode, calculation, and mapping options for Circuitscape were set to pairwise mode (run in low‐memory mode), use average conductance instead of resistance for connections between cells, write cumulative and max current maps only, and set focal node currents to zero (Dai, Hacker, et al., 2019). We inverted the RI value to link the risk region of house break‐ins with low movement resistance, and vice versa. Specifically, we used the functions of negative exponential transformation to convert RI into resistance values (Dai, Hacker, et al., 2019; Keeley, Beier, & Gagnon, 2016; Li et al., 2018):

$$\begin{array}{c} \text{If RI}>\text{Threshold}\to \text{Risk Region}\to \text{Resistance}=1\\ \text{If RI}<\text{Threshold}\to \text{Nonrisk Region}\to \text{Resistance}=e^{\frac{\ln(0.001)}{\text{threshold}}\times \text{RI}}\times 1,000 \end{array}$$

## 3 RESULTS

### 3.1 Model performance

**Figure 3:**
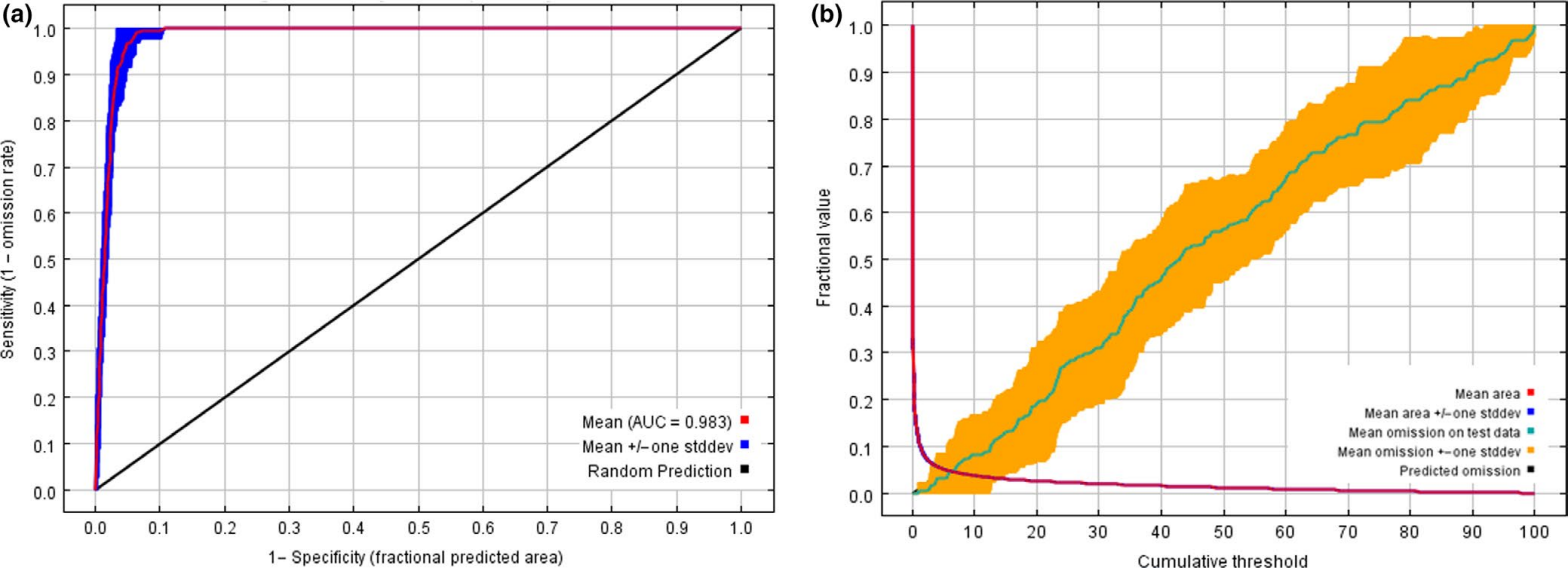
Statistical graphs of MaxEnt model output results. (a) is the receiver operating characteristic (ROC) curve and average test AUC for accuracy analysis of risk prediction by MaxEnt model and (b) is the analysis of test omission rate and predicted area, where values indicate the training gain only with variables

In the MaxEnt model, 218 occurrence points and 11 variables were used to construct the risk assessment model. The percent contribution of model variables ranked from highest to lowest were Land use type (34.1%), Population density (31.4%), Normalized Difference Vegetation Index (10.4%), Distance to rivers (10.1%), Distance to lakes; (4.8%), Distance to Sanjiangyuan National Park (3.9%), Density of lake distribution (2.7%), Elevation (2%), Slope (0.4%), Density of river distribution (0.2%), and Aspect (0.1%). Land use type was found to have the greatest influence on the spatial risk distributions of house break‐ins. The cross‐validation value illustrated sufficient performance for model outputs (average testing AUC was 0.983 ± 0.0042; average training AUC was 0.986 ± 0.0001; Figure 3).

### 3.2 Risk distributions of house break‐ins

**Figure 4:**
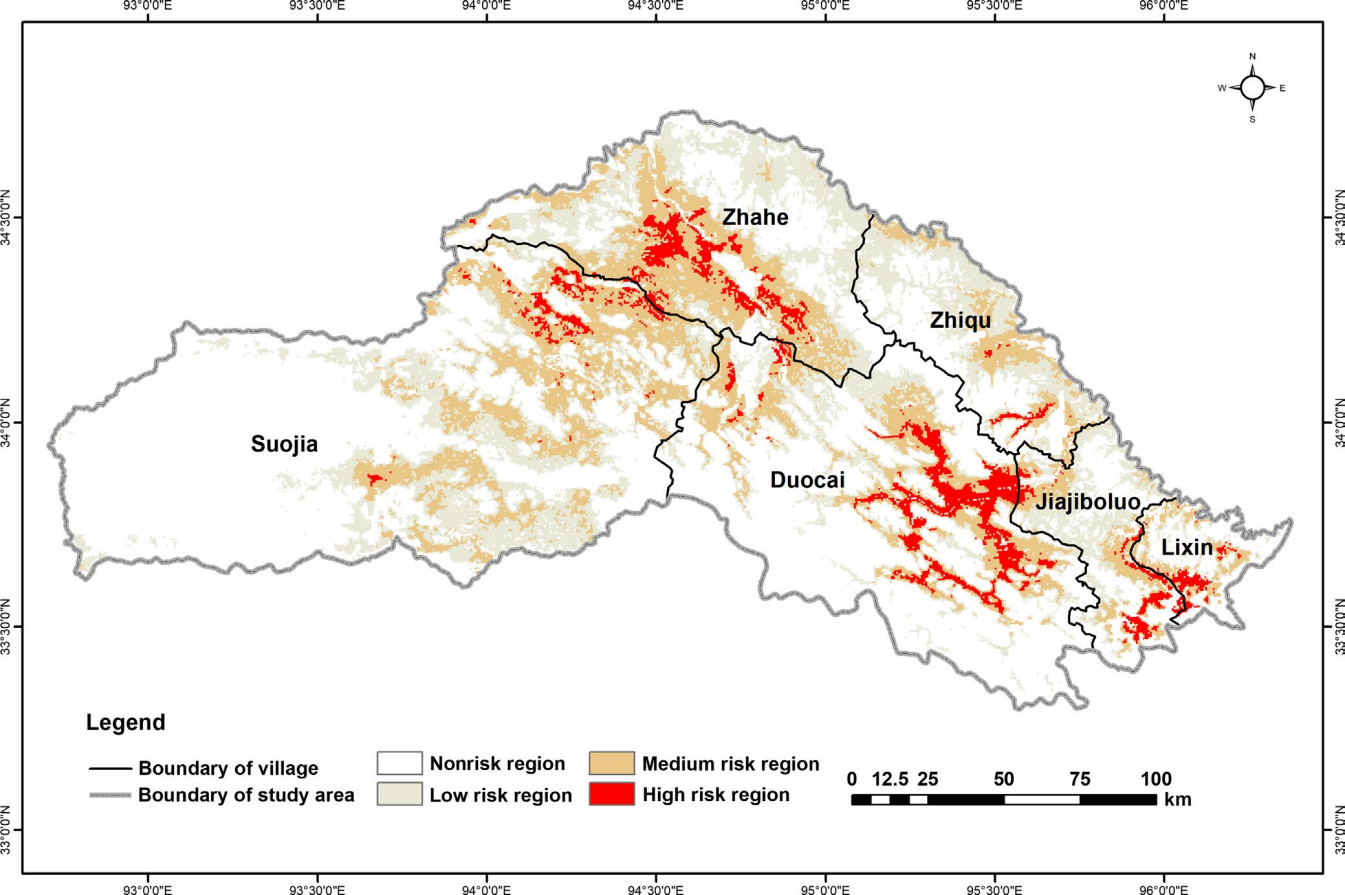
Predicted risk areas of house break‐ins caused by brown bears in Zhiduo County of the Sanjiangyuan region

The total risk area of house break‐ins caused by brown bears was 11,577.91 km^2^, accounting for 29.85% of Zhiduo County of the Sanjiangyuan region (Table 1 and Figure 4). The low‐risk area covered the largest portion of land (about 5,633.01 km^2^), accounting for 48.65% of the total risk area, followed by medium risk (4,811.66 km^2^, 41.56%) and high risk (1,133.24 km^2^, 9.79%; Table 1). Suojia had the largest risk area (3,950.94 km^2^; 34.12%), followed by Zhahe (2,800.43 km^2^; 24.19%), Duocai (2,471.10 km^2^; 21.34%), Zhiqu (1,174.09 km^2^; 10.14%), Jiajiboluo (884.42 km^2^; 7.64%), and Lixin (296.93 km^2^; 2.56%; Table 1 and Figure 4).

**Table 1:** Statistics of risk region of house break‐ins caused by brown bears in Zhiduo County of the Sanjiangyuan region (area unit: km^2^; percent unit: %)

Township	Nonrisk	Low risk		Medium risk		High risk		Total of risk	
		Area	Percent	Area	Percent	Area	Percent	Area	Percent
Suojia	12,095.38	2,075.3	52.53	1,734.12	43.89	141.52	3.58	3,950.94	34.12
Zhahe	3,548.08	1,307.41	46.69	1,206.68	43.09	286.34	10.22	2,800.43	24.19
Duocai	7,300.32	899.03	36.38	1,059.22	42.86	512.85	20.75	2,471.10	21.34
Zhiqu	1,937.47	771.23	65.69	372.93	31.76	29.93	2.55	1,174.09	10.14
Lixin	850.28	87.29	29.4	153.00	51.53	56.64	19.08	296.93	2.56
Jiajiboluo	1,483.96	492.75	55.71	285.71	32.3	105.96	11.98	884.42	7.64
Total	27,215.49	5,633.01	48.65	4,811.66	41.56	1,133.24	9.79	11,577.91	100

**Figure 5:**
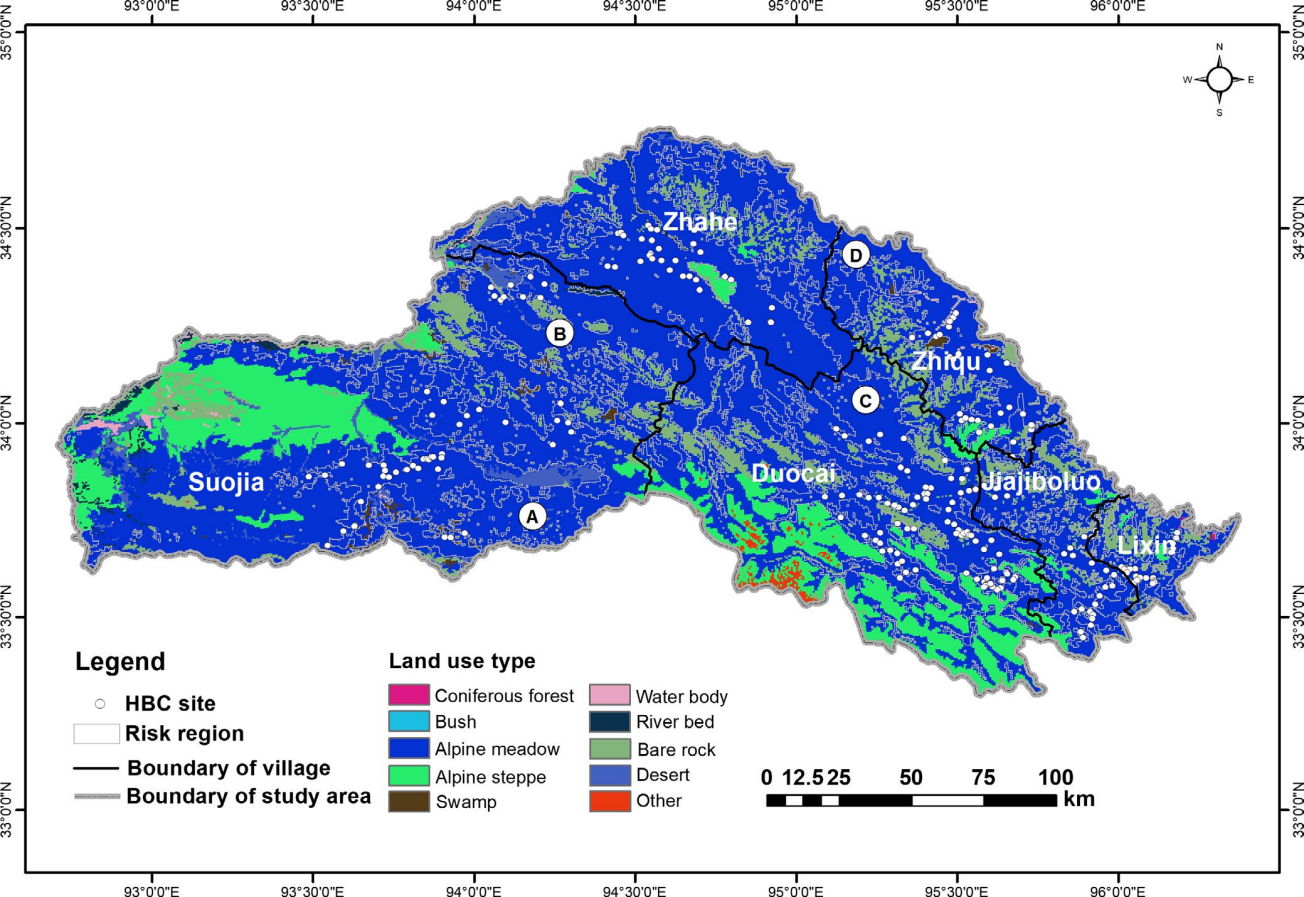
The land use type of house break‐in risk areas

Predicted risk areas occurred widely from southeast to northwest in the study area and most were distributed in Suojia and Zhahe (6,751.37 km^2^), located in the Yangtze River Zone of Sanjiangyuan National Park, accounting for 58.31% of the total risk area (Table 1 and Figure 4). However, Duocai, located outside the Sanjiangyuan National Park, had the largest proportion of high‐risk area (512.85 km^2^), accounting for 45.26% of the total high‐risk area. By analysis of types of land use in risk regions, it found that the area of the alpine meadow is the largest at 11,060.34 km^2^, accounting for 95.53% of the total risk area (Figure 5; Appendix S2).

### 3.3 The risk diffusion paths of brown bears

**Figure 6:**
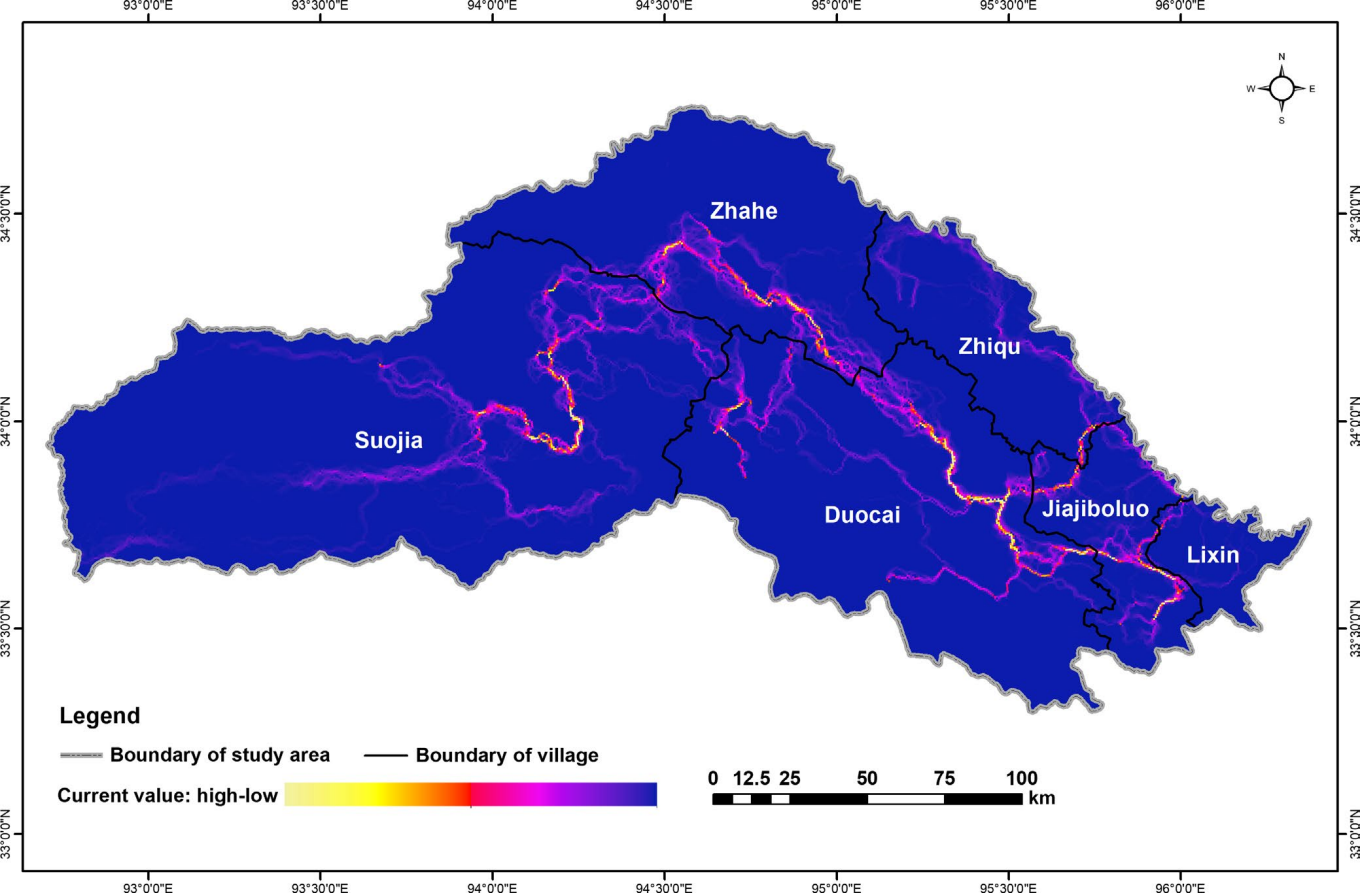
Risk diffusion paths for brown bears in Zhiduo County were simulated by the Circuit model based on the risk map

Eastern Suojia, southern Zhahe, eastern Duocai, and southern Jiajiboluo exhibited risk diffusion paths with high current flow extending from the southeast to the northwest, connecting the inside and outside of the Sanjiangyuan National Park (Figure 6). Risk diffusion paths with high current flow were primarily distributed in high‐ and medium‐risk areas, whereas risk diffusion paths with low current flow were primarily located in low‐risk areas (Figures 4 and 6).

## 4 DISCUSSION

### 4.1 Model limitations

The results of this study have some limitations and uncertainties. First, some factors related to human–bear encounter probability were missing, such as herders' grazing range. We initially planned to use participatory mapping to outline herders' grazing ranges across seasons, but the difference in summer pasture from year to year made it impossible to unify and standardize the resulting data. As an alternative, we contacted the local Animal Husbandry Bureau for information on pasture range size, but spatial distribution maps of ranges were unavailable. Another limitation was inability to interview households in less accessible habitat. Zhiduo County is located in the hinterland of the Qinghai–Tibet Plateau. This area is characterized by high altitudes, harsh weather, and limited roadways, with its inhabitants living vast distances between each other. There likely are occurrence points of house break‐ins caused by brown bears that were not collected due to these research challenges, which may bias results. Nonetheless, outcomes represent a reliable analysis of the risk distribution areas of house break‐ins caused by brown bears and are based on the best available data.

### 4.2 Representative type of brown bear damage

Tibetan brown bears have been the most dangerous species for humans in the Sanjiangyuan region (Dai, Li, et al., 2019; Dai, Xue, et al., 2019; Wu, 2014). They not only predate livestock, but also break into houses and attack people (Dai, Li, et al., 2019; Dai, Xue, et al., 2019; Han et al., 2018; Wu, 2014), a behavior which herders find intolerable (Dai, Xue, et al., 2019). In our field survey, we found that house break‐ins by brown bears posed the greatest threat to the livelihood of local people. However, the type of brown bear damage in other areas of the Himalayas, such as Nepal, is dominated by livestock depredation, rather than house break‐ins (Aryal, Hopkins, et al., 2012). Brown bears raiding houses in the Sanjiangyuan region of China may be directly related to the availability of accessible food (Dai, Xue, et al., 2019). Herders in the Sanjiangyuan region store food in their permanent winter houses over the summer while they are away (Dai, Li, et al., 2019; Dai, Xue, et al., 2019). Over time, brown bears have learned to exploit this food resource (Dai, Xue, et al., 2019). Hibernation patterns may also play a role. Tibetan brown bears hibernate from late October or early November to the middle of March of the following year (Han et al., 2018). Post hibernation, brown bears need to replenish energy, but the temperature in the Sanjiangyuan region is still very low and most marmots have not finished hibernation, causing a natural food shortage. Therefore, brown bears seek high‐energy food in herders' homes, such as yak butter, flour, meat, and livestock fodder (Dai, Xue, et al., 2019). In the process of searching for food, daily supplies (e.g., cabinets, teapots, bowls, and calfskin bags) are damaged, resulting in even more economic loss (Dai, Xue, et al., 2019).

### 4.3 Characteristics of risk distributions

#### 4.3.1 Spatial variation of risk distributions

More risk areas for house break‐ins were found inside as opposed to outside Sanjiangyaun National Park (Figure 4). This may be related to a larger population of brown bears in the national park. Another considerable factor is the national park's rich biodiversity. Although national parks offer some degree of refuge to brown bears from human disturbance and are vital for conservation, many have insufficient resources to harbor large populations of brown bears, encouraging brown bears to seek readily available food sources in areas with herder settlements. However, some risk areas were geographically distant from the Sanjiangyuan National Park, indicating that brown bears may be incentivized to travel further if nearby winter houses lack food. For instance, when herders move to their summer pasture, brown bears may be prone to approach regions where herders are occupying temporary living spaces (Dai, Xue, et al., 2019). The third contributing factor could be that a proportion of the herders' activity range in the Sanjiangyuan National Park overlaps with brown bears' habitat (Dai, Xue, et al., 2019).

There was lower risk in the western region of Suojia and southwestern of Duocai (Figure 4). This may be due to fewer brown bears in the area given the reduced area of suitable habitat and food in these regions. Alpine steppe habitat is less suitable than alpine meadow for brown bears (Wu, 2014; Xu et al., 2006), and the west regions of Suojia and southwestern of Duocai have fewer continuous alpine meadow patches than that in other regions of study area (Figure 5; Appendix S2). Further, alpine steppe patches (Figure 5; Appendix S2) harbor fewer pikas and marmots.

The southeastern (A) and northeastern areas (B) of Suojia, the northern areas (C) of Duocai, and northwestern areas (D) of Zhiqu had higher risk index (Figure 5), but currently there are no records of Tibetan brown bears breaking into houses or preying on livestock in these regions. However, these areas constitute continuous alpine meadow patches and are relatively close to other present bear damage sites (Figure 5), suggesting a greater potential likelihood of future brown bear raiding. Protective measures (e.g., electric or barrier fences) for local settlements in these regions should be taken in advance.

#### 4.3.2 The direction of risk diffusion paths

The risk diffusion paths extended from the southeast to the northwest, connecting the inside of Sanjiangyuan National Park to the outside. This direction of risk diffusion paths is possibly related to the fact that human disturbance within the Sanjiangyuan National Park is more scarce compared to the outer region of the national park. It has been noted that the habitat quality within the Sanjiangyuan National Park is much higher than that of the national park exterior; therefore, areas within Sanjiangyuan National Park provide an ideal refuge for brown bears (Dai, Xue, et al., 2019). In addition, there are more herders' homes outside of the Sanjiangyuan National Park than within the national park, thus providing more readily available and high‐energy food sources for brown bears. Upon both the onset and completion of hibernation, brown bears require substantial amounts of high‐energy fatty foods. Because these foods are lacking in Sanjiangyuan National Park (Wu, 2014), brown bears may diffuse along these paths to more human dominated settlements in search of human food, such as dairy products, meat products, and livestock (Dai, Xue, et al., 2019).

The risk diffusion paths of Zhiqu, Jiajiboluo, and Lixin spread to the Sanjiangyuan National Park through Duocai; the risk diffusion paths of Suojia and Zhahe spread out to the Sanjiangyuan National Park through Duocai (Figure 6). Duocai serves as a core connection point for risk diffusion paths inside and outside the national park. This may make herders living in these areas more susceptible to the negative outcomes of bear damage. It is essential to implement and strengthen protection measures in these areas.

The high current diffusion paths were mainly concentrated on alpine meadows (Figure 5; Figure 6). Alpine meadows provide rich natural food for the brown bear during the migration. Herder settlements are at a low density inside the limits of the Sanjiangyuan National Park, so bears traveling through the area rely on natural, rather than human food for energy. Alpine meadows are richer in rodents than other types of vegetation; therefore, brown bears tend to choose alpine meadow patches when they spread over long distance.

### 4.4 Suggestions to mitigate bear damage

#### 4.4.1 Conventional and new mitigation strategies to mitigate bear damage

Local herders have adopted a variety of strategies to discourage brown bears from breaking into their homes. These include dogs, scarecrows, firecrackers, strengthening doors and walls, and building fences around homes (Dai, Xue, et al., 2019; Han et al., 2018; Wu, 2014). When families move to their summer pastures, economic losses that could be caused by brown bears to their winter homes can be decreased in a number of ways. These include placing 24‐hr solar‐powered radios in winter homes to create the illusion that the house is inhabited, leaving doors and windows open, carrying all food with them when moving to summer pastures, asking relatives to keep watch of their houses, and putting iron nail plates around houses (Dai, Xue, et al., 2019; Han et al., 2018). We suggest some additional methods to be adopted by the local government and neighboring wildlife agencies to prevent bear damage, such as electric fences (Ambarli & Bilgin, 2008; Huygens & Hayashi, 2000; Proctor et al., 2018; Sapkota, Aryal, Baral, Hayward, & Raubenheimer, 2014), steel bins (Schirokauer & Boyd, 1998), bear spray (Miller, Freimund, Metcalf, Nickerson, & Powell, 2019; Smith, Herrero, Debruyn, & Wilder, 2011), and diversionary feeding (Kubasiewicz, Bunnefeld, Tulloch, Quine, & Park, 2015). These measures should first consider herders who are located in high‐risk areas or in proximity to high current risk diffusion paths. Mitigation measures should also be tailored to region, taking into account local realities such as geography, laws and regulations, culture and religious practices (Dai, Li, et al., 2019; Dai, Xue, et al., 2019).

#### 4.4.2 Strengthen management of the high current risk diffusion paths

Although the herders living around high current risk diffusion paths, such as the herders in eastern region of Duocai (Figure 6), are more likely to suffer brown bear damage, it remains that these risk diffusion paths serve as important ecological corridors for brown bears and must be protected. The disruption of bear migration would have negative impacts on local ecosystems and human life. For example, if unable to migrate to resource‐rich areas, brown bears may seek at herder homes for food at even higher rates. In addition, reduced accessibility between populations previously provided by corridors would reduce genetic exchange inside and outside the national park. In order to reduce the property losses of herders living around the high current risk diffusion paths, local government and wildlife responsible agencies should focus on strengthening the protection of their properties. At the same time, warning signs should be placed around the high current risk diffusion paths, and humans should always be alert to the presence of brown bears.

#### 4.4.3 Compensation suggestions based on predicted risk map

Financial compensation programs play an important role in supporting herders that live in carnivore damage hotspots within national park boundaries (Aryal et al., 2014). In Sanjiangyuan National Park, the compensation program is key to minimizing retaliation against carnivores (Dai, Li, et al., 2019; Dai, Xue, et al., 2019). Compensation programs also present opportunities for pastoral communities to establish close relationships with the local Forestry Department, engendering trust in authority that can improve attitudes toward conservation (Dickman, 2010; Treves & Karanth, 2010; Miller et al, 2015). However, only one compensation program currently supports herders in the Sanjiangyuan National Park, and this cannot fully resolve carnivore damages (Li et al., 2018; Morehouse, Tigner, & Boyce, 2018; Nyhus, Fischer, Madden, & Osofsky, 2003). In conjunction with the current compensation program, the local government should purchase insurance for herders' properties to supplement compensation from the destruction of homes and loss of livestock. High, medium and low‐grade insurance should be purchased in accordance to the high, medium, or low‐risk area where herders are living.

## 5 CONCLUSION

Assessing risk areas and simulating risk diffusion paths are crucial steps toward mitigating brown bear damage and provide a foundation for developing conservation programs and policies that aimed at making coexistence possible. We used maximum entropy (MaxEnt) model to assess the risk regions of house break‐ins caused by Tibetan brown bears and to predict future risks. We then used the circuit theory model based on the random walk theory to simulate risk diffusion paths of brown bears within Zhiduo County of the Sanjiangyuan region. To foster human–bear coexistence, we suggest that local government and wildlife agencies focus on managing risk areas and risk diffusion paths of brown bears, and work toward implementing new mitigation strategies, such as electric fences, steel bins, bear spray, and diversionary feeding. We also propose some compensation suggestions based on our predicted risk map. We hope our analytical methods can be applied to carnivore damage reduction efforts and carnivore conservation planning across the Qinghai–Tibet Plateau.

## CONFLICT OF INTEREST

None declared.

## AUTHOR CONTRIBUTIONS

Diqiang Li and Yadong Xue developed concept and led manuscript production. Yunchuan Dai performed the structure of manuscript, and drafted the first version of the manuscript. Charlotte E. Hacker, Yuguang Zhang, Wenwen Li, Jia Li, and Haodong Liu led spatial modeling and contributed to manuscript writing. Yu Zhang, Gongbaocairen Bona and Ye Li performed the data collection and data analysis. All co‐authors participated in the scientific discussions and commented on the manuscript.

### OPEN RESEARCH BADGES


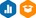
This article has been awarded Open Materials and Open Data Badges. All materials and data are publicly accessible via the Open Science Framework at https://doi.org/10.5061/dryad.brv15dv5m.

## Data Availability

All authors agreed to deposit data from this manuscript to a public repository. Data are submitted to Dryad, and DOI number is https://doi.org/10.5061/dryad.brv15dv5m.
