# Supplementary material for: Identifying the risk regions of house break‐ins caused by Tibetan brown bears (Ursus arctos pruinosus) in the Sanjiangyuan region, China
Source: Ecol Evol. 2019 Dec 8;9(24):13979–90. doi: 10.1002/ece3.5835 (PMC6953560; doi:10.1002/ece3.5835)
Supplement: Supplementary file 1 [file ECE3-9-13979-s001.doc]

**Appendix 1** Correlation coefficient of environmental variables

| Layers | ELE | SLO | ASP | HPD | HII | LUT | NDVI | DSS | DSR | DRD | DSL | DLD |
| --- | --- | --- | --- | --- | --- | --- | --- | --- | --- | --- | --- | --- |
| ELE | 1.0000 |  |  |  |  |  |  |  |  |  |  |  |
| SLO | 0.1286 | 1.0000 |  |  |  |  |  |  |  |  |  |  |
| ASP | 0.0226 | 0.0247 | 1.0000 |  |  |  |  |  |  |  |  |  |
| HPD | -0.1827 | 0.3636 | 0.0082 | 1.0000 |  |  |  |  |  |  |  |  |
| HII | -0.3383 | 0.3083 | 0.0055 | 0.6094 | 1.0000 |  |  |  |  |  |  |  |
| LUT | -0.3343 | -0.0934 | -0.0191 | -0.2511 | -0.0218 | 1.0000 |  |  |  |  |  |  |
| NDVI | -0.0215 | 0.3175 | -0.0207 | 0.5653 | 0.5384 | -0.2522 | 1.0000 |  |  |  |  |  |
| DSS | -0.1838 | 0.0238 | -0.0060 | 0.2129 | 0.1248 | -0.3889 | 0.0598 | 1.0000 |  |  |  |  |
| DSR | 0.1916 | -0.2136 | -0.0138 | -0.3021 | -0.4296 | -0.0195 | -0.4041 | -0.0502 | 1.0000 |  |  |  |
| DRD | -0.1182 | 0.2594 | 0.0118 | 0.3474 | 0.4639 | 0.0188 | 0.4456 | -0.0679 | -0.5880 | 1.0000 |  |  |
| DSL | -0.1474 | 0.3267 | 0.0196 | 0.4314 | 0.3790 | -0.1187 | 0.4632 | 0.0712 | -0.2587 | 0.2101 | 1.0000 |  |
| DLD | 0.0794 | -0.1338 | -0.0002 | -0.1448 | -0.0977 | 0.0052 | -0.0714 | -0.0638 | -0.0763 | 0.1612 | -0.1991 | 1.0000 |

ELE: elevation, SLO: slope, ASP: aspect, HPD: human population density, HII: human influence index, LUT: land use type, NDVI: normalized difference vegetation index, DSS: distance to the Sanjiangyuan National Park, DSR: distance to rivers, DRD: density of river distribution, DSL: distance to lakes; DLD: density of lake distribution.

**Appendix 2** Statistics of land use type in different risk region (area unit: km^2^)

| Land use type | Suojia | Zhahe | Duocai | Zhiqu | Lixin | Jiajiboluo | Percentage |
| --- | --- | --- | --- | --- | --- | --- | --- |
| Coniferous forest | － | － | － | － | 0.86 | 0.21 | 0.01 % |
| Bush | － | － | 1.17 | － | 2.88 | 0.45 | 0.04 % |
| Alpine meadow | 3,797.90 | 2,721.69 | 2,386.32 | 1,119.57 | 257.18 | 777.68 | 95.53 % |
| Alpine steppe | 3.49 | 5.69 | 3.10 | 0.12 | 0.36 | 0.16 | 0.11 % |
| Swamp | 35.94 | 2.33 | 1.86 | 7.39 | 0.53 | 2.09 | 0.43 % |
| Water body | 1.76 | 1.63 | － | 14.73 | 0.95 | 1.47 | 0.18 % |
| River bed | 10.66 | 26.33 | 1.87 | 1.80 | 1.00 | 2.34 | 0.38 % |
| Bare rock | 25.61 | 31.22 | 73.22 | 29.17 | 33.17 | 94.26 | 2.48 % |
| Desert | 75.58 | 11.54 | 3.56 | 1.31 | － | 5.76 | 0.84 % |
